# Unresected small lymph node assessment predicts prognosis for patients with pT3N0M0 thoracic esophageal squamous cell carcinoma

**DOI:** 10.1186/s12957-021-02412-1

**Published:** 2021-10-18

**Authors:** Yi Wang, Ping Xiao, Ningjing Yang, Xiang Wang, Ke Ma, Lei Wu, Wei Zhang, Xiang Zhuang, Tianpeng Xie, Qiang Fang, Mei Lan, Qifeng Wang, Lin Peng

**Affiliations:** 1 Department of Radiation Oncology, Sichuan Cancer Hospital and Institution, Sichuan Cancer Center, School of Medicine, University of Electronic Science and Technology of China, Radiation Oncology Key Laboratory of Sichuan Province, No.55,Section 4,South Renmin Road, Chengdu, 610042 China; 2 Department of Medical Oncology, Sichuan Cancer Hospital and Institution, Sichuan Cancer Center, School of Medicine, University of Electronic Science and Technology of China, Chengdu, China; 3 Department of Thoracic Surgery, Sichuan Cancer Hospital and Institution, Sichuan Cancer Center, School of Medicine, University of Electronic Science and Technology of China, Chengdu, China; 4 Department of Radiology, Sichuan Cancer Hospital and Institution, Sichuan Cancer Center, School of Medicine, University of Electronic Science and Technology of China, Chengdu, China; 5 Department of PET/CT center, Sichuan Cancer Hospital and Institution, Sichuan Cancer Center, School of Medicine, University of Electronic Science and Technology of China, Chengdu, China

**Keywords:** Esophageal cancer, Lymph node assessment, Prognostic factor, Postoperative adjuvant therapy, Unresected small lymph nodes, Computed tomography

## Abstract

**Background:**

The role of unresected small lymph nodes (LNs) which may contain metastases for thoracic esophageal squamous cell carcinoma (TESCC) has not been addressed. The aim of the study was to investigate the role of unresected small LNs assessment using computed tomography (CT) in prognostic estimates of pT3N0M0 TESCC patients.

**Methods:**

Between January 2009 and December 2017, 294 patients who underwent esophagectomy with R0 resection at Sichuan Cancer Hospital were retrospectively examined, and the last follow-up time was July 2018. Patients were classified into CT-suspect and CT-negative groups according to the shortest diameter and the shape (axial ratio) of the unresected small LNs on preoperative CT. The Kaplan–Meier method was used to compare survival differences in prognostic factors. Univariate and multivariate analyses were performed to identify prognostic factors for survival and recurrence.

**Results:**

Eighty-four patients (28.6%) were classified as CT-suspect group according to the diagnostic criteria; survival analysis suggested that CT-suspect group of patients had a relatively poorer prognosis (*P*<0.05). Cox regression analysis indicated that unresected small LNs status, tumor grade, and postoperative adjuvant therapy were independent prognostic factors for patients with pT3N0M0 TESCC (*P*<0.05). Further analysis shown the rates of total recurrence (TR) and locoregional recurrence (LR) in the CT-suspect group were significantly higher than that in the CT-negative group (TR, *P*<0.001; LR, *P*<0.001). Among the LRs, the rate of supraclavicular lymph node recurrence in the CT-suspect group was significantly higher than that in the CT-negative group (*P*<0.001).

**Conclusions:**

Unresected small lymph node assessment is critically important and predict prognosis for pT3N0M0 TESCC patients.

**Supplementary Information:**

The online version contains supplementary material available at 10.1186/s12957-021-02412-1.

## Background

Esophageal cancer (EC) is an aggressive disease with a poor prognosis and high mortality rate globally [1, 2]. In China, EC is the sixth most common cancer and the fourth leading cause of cancer-related death. More than 90% of ECs are pathologically diagnosed as esophageal squamous cell carcinoma (ESCC) [3–5]. The status of lymph nodes (LNs) has been considered as the most critical prognostic factor affecting long-term survival of patients with ESCC [6]. For pathological T3 stage patients, there is a high possibility of LN metastasis, as the likelihood of LN metastasis occurs with increasing T stage [7]. Thus, for patients with pathological T3 stage and pathological N0 status, there is a high possibility of metastatic nodes to be present among unresected LNs when all removed LNs are confirmed to be pathologically cancer-negative.

Therefore, precise evaluation of these unresected LNs is the key to estimate the prognosis of patients. CT is the most commonly used non-invasive method to evaluate metastatic infiltration of lymph nodes in EC. Traditionally, LNs with a short diameter of ≥10 mm on CT were considered to be metastases [8, 9]. Thus, only patients with unresected LNs in a short-axis diameter greater than 10 mm are classified as having undergone R2 resection according to “The Pathologist and the Residual Tumor (R) Classification” [10, 11]. Patients with unresected regional LNs in a short-axis diameter smaller than 10 mm would still be treated as having regional lymph nodes completely removed. However, studies have suggested that only 8.0–37.5% of metastatic lymph nodes in EC were greater than 10 mm [12, 13]. Evidently, there is a possibility that the residual tumor (R) classification may underestimate the risk because of the inaccuracy of CT diagnostic criteria of lymph node metastasis.

In addition, studies have reported that lymph nodes contain metastasis tend to be round, with the lymph node axial ratio (short-axis diameter/long-axis diameter) approaching “1” [14, 15]. Previously study has demonstrated that the combination of a smaller size and axial ratio for LNs in CT as criteria improves the detection sensitivity for LN metastases in EC [16]. Hence, we aimed to observe whether distinguishing unresected small LNs (short diameters of <10 mm) based on such criteria can affect prognosis in a homogeneous cohort of patients who underwent radical (R0) resection for T3N0M0 TESCC.

## Methods

### Eligibility

The medical records of patients with pT3N0M0 TESCC who were treated at the Sichuan Cancer Hospital between January 2009 and December 2017 were retrospectively reviewed. Patients with confirmed pT3N0M0 TESCC according to the 8th edition of the American Joint Committee on Cancer (AJCC) Tumor–Node–Metastasis (TNM) staging system who underwent initial transthoracic subtotal esophagectomy plus lymphadenectomy with R0 resection were included. The exclusion criteria were (1) no CT records before surgery, (2) no postoperative CT records within 6 months postoperatively, (3) R1 or R2 surgery (including unresected LNs with short diameters of ≥10 mm), (4) loss to follow-up within 3 months of surgery, (5) history of neoadjuvant therapy, or (6) death within 3 months of surgery. This study was approved by the ethics committee of our institution with ethical item number of SCCHEC-02-2020-015. Informed consent was exempted by the ethics committee.

### Surgery

The surgical approach and procedure were determined based on the tumor’s location and the surgeon’s preference. The surgical approaches were standard McKeown esophagectomy with at least two-field (thoracic and abdominal) lymphadenectomy or Ivor–Lewis esophagectomy with two-field (thoracic and abdominal) lymphadenectomy. Radical surgical resection consisted of a transthoracic subtotal esophagectomy on the right side, including abdominal, mediastinal, and even cervical lymphadenectomy.

### Adjuvant therapy

Since the standard for postoperative adjuvant therapy in patients with EC is controversial, the selection of postoperative adjuvant chemotherapy (POCT) or adjuvant chemoradiotherapy/radiotherapy (aCRT/RT) was based on the physician’s preference and general physical condition of the patient. Cisplatin-based chemotherapy was the most commonly used agent in POCT; the median number of chemotherapy cycles was 3 (range, 1–6). Intensity-modulated radiotherapy (IMRT) was used for patients who received aCRT/RT. The total dose for aCRT/RT was 45–60 Gy, and the daily fraction dose was 1.8–2.0 Gy.

### Pre- and postoperative CT examination

All patients underwent enhanced neck, thorax, and upper abdomen CT scanning within 2 weeks before surgery and within 6 months postoperatively. CT was performed with a 64-row helical CT scanner (General Electrical Medical Systems, Milwaukee, WI, Lightspeed VCT) in the crania–caudal direction starting from the neck to the renal hilum level with a slice thickness of 3 mm.

### Assessment of unresected small lymph nodes

**Fig. 1:**
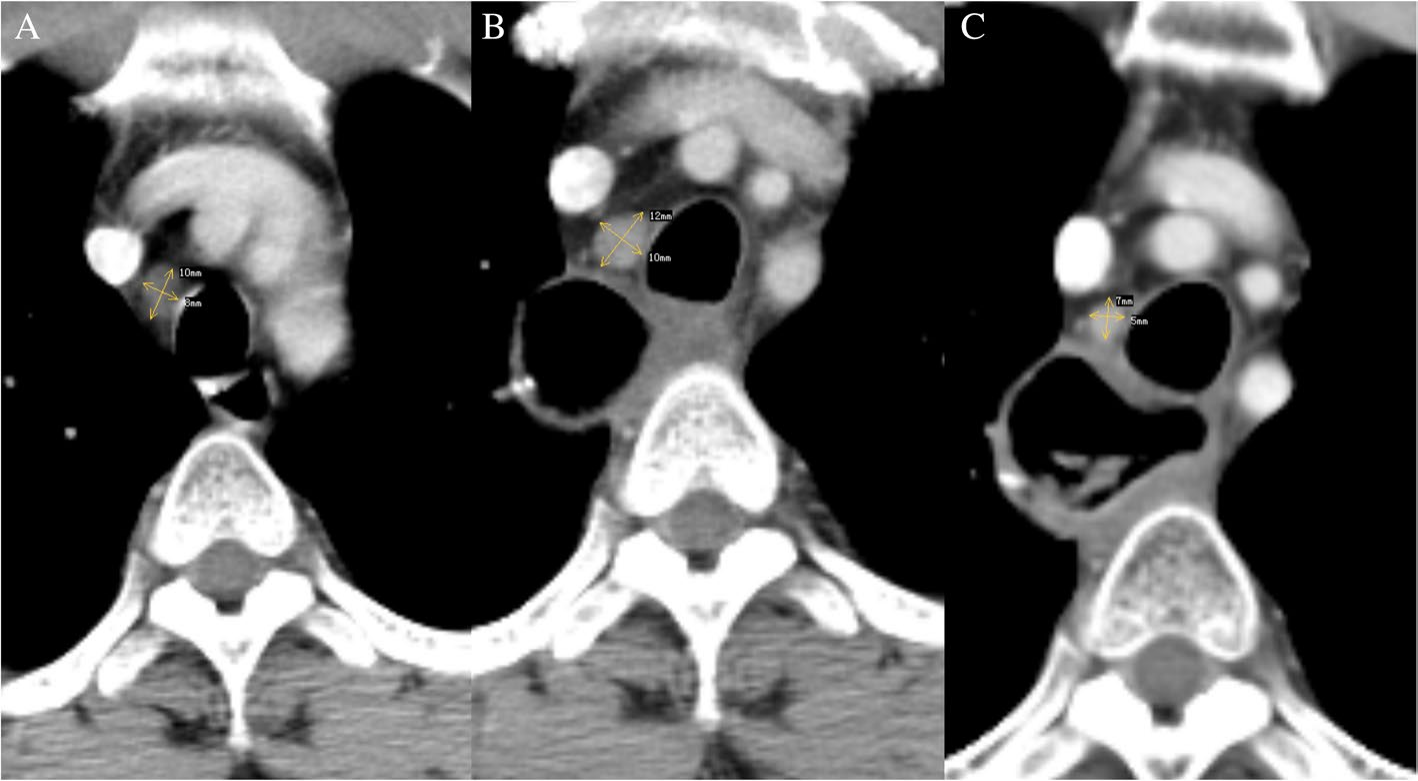
Contrast-enhanced CT image of a 47-year-old man shows a small lymph node in the right upper paratracheal. The short and long diameters are 8 mm and 10 mm in the transverse section on the preoperative CT, with an axial ratio of 0.8 (8/10) (**A**), this small lymph node was confirmed unresected on postoperative CT 2 months later, with a larger size of 10×12mm in the transverse section (**B**), and shrunk after radiotherapy with a size of 5×7 mm in the transverse section (**C**)

CT images were analyzed on a PACS station by two independent radiologists with at least 8 years’ experience, who blinded to clinical and histopathologic information. Unresected LNs were defined as regional LNs that were found on preoperative CT but still existed on postoperative CT, independent of their size on postoperative CT. Unresected small LNs were diagnosed as clinically suspected metastatic LNs if they had the shortest diameter exceeding 5 mm in the soft-tissue window and the shape of the nodes were round (axial ratio exceeding 0.66) in transverse section on preoperative CT (Fig. 1), and the rest of unresected small LNs were considered to be negative LNs (the size change of CT-suspect metastatic unresected small lymph nodes in different conditions are shown in Appendix (Table 1)). According to the unresected small LNs status, all patients were divided into two groups: CT-suspect group and CT-negative group.

### Follow-up

Patients were assessed weekly during treatment. Follow-up was scheduled every 3–6 months for the first 2 years after treatment, every 6–12 months for the following 3 years, and annually thereafter. All relapses were confirmed using CT, magnetic resonance imaging, or endoscopy at the corresponding sites. Cytology or histology was performed if necessary. Total recurrence (TR), locoregional recurrence (LR), distant metastasis (DM), and overall survival (OS) were analyzed in this study. Specifically, TR was defined as any recurrence or metastasis during the follow-up period. LR was defined as any locoregional tumor recurrence and/or metastatic lymph node at cervical, mediastinal, and upper abdomen regions defined by the 8th edition of the AJCC TNM staging system. DM was defined as any event of recurrence or metastasis other than LR. OS was measured from the date of operation to the date of death or last follow-up and was censored at the last contact date in surviving patients.

### Statistical analysis

A chi-square test was used to compare categorical data, with or without correction for continuity. Actual survival was calculated and compared between groups using the Kaplan**–**Meier method and log-rank test, respectively. Univariate and multivariate analyses of the prognostic factors were performed using the log-rank test and Cox regression model, respectively. A confidence value of 95% (*P*<0.05) was considered significant. SPSS 22.0 for Windows was used for statistical analyses.

## Results

### Clinical data

A total of 294 patients with pT3N0M0 TESCC were eligible for this analysis with a median age of 61 years (range, 38–83), including 228 men (77.6%) and 66 women (22.4%). Regarding tumor location, 70 tumors (23.8%) were in the upper thoracic region, 163 (55.4%) in the middle thoracic region, and 61 (20.8%) in the lower thoracic region. Of these, the McKeown procedure was performed in 202 patients (68.7%), and 92 patients (31.3%) underwent the Ivor–Lewis procedure. The median length of the lesion was 4 cm (range, 1–10 cm). The median number of LNs removed was 20 (range, 1–55). A total of 63 patients (21.4%) had well-differentiated (G1) tumors, 136 (46.3%) had moderately differentiated (G2) tumors, and 95 (32.3%) had poorly differentiated (G3) tumors. A total of 139 patients (47.3%) underwent surgery alone, 132 (44.9%) underwent surgery with POCT, 5 (1.7%) underwent surgery with RT, and 18 (6.1%) underwent surgery with aCRT. The patients’ demographic data are shown in Table 1. The median follow-up duration was 32 months (range, 1.7–104.8), with 3- and 5-year survival rates of 69.3%, and 61.1% for the overall study group, respectively.

**Table 1:** General characteristics of patients with pT3N0M0 stage thoracic esophageal squamous cell carcinoma

Characteristic	Total (*n*=294)	CT-suspect group (*n*=84)	CT-negative group (*n*=210)	*χ*^2^	*P* value
Sex				3.284	0.070
Male	228 (77.6%)	71 (84.5%)	157 (74.8%)		
Female	66 (22.4%)	13 (15.5%)	53 (25.2%)		
Age (years)				0.721	0.396
<60	141 (48.0%)	37 (44.0%)	104 (49.5%)		
≥60	153 (52.0%)	47 (56.0%)	106 (50.5%)		
Tumor location				0.411	0.814
Upper thoracic	70 (23.8%)	19 (22.6%)	51 (24.3%)		
Middle thoracic	163 (55.4%)	49 (58.3%)	114 (54.3%)		
Lower thoracic	61 (20.8%)	16 (19.0%)	45 (21.4%)		
Surgical approach				1.723	0.189
Ivor–Lewis	92 (31.3%)	31 (36.9%)	61 (29.0%)		
McKeown	202 (68.7%)	53 (63.1%)	149 (71.0%)		
Length of lesion				0.311	0.577
<4 cm	171 (58.2%)	39 (46.4%)	90 (42.9%)		
≥4 cm	123 (41.8%)	45 (53.6%)	120 (57.1%)		
Angioinvasion				0.707	0.400
No	272 (92.5%)	76 (90.5%)	196 (93.3%)		
Yes	22 (7.5%)	8 (9.5%)	14 (6.7%)		
Perineural invasion				0.973	0.324
No	56 (19.0%)	65 (77.4%)	173 (82.4%)		
Yes	238 (81.0%)	19 (22.6%)	37 (77.4%)		
No. of removed LNs				1.875	0.171
<15	88 (29.9%)	30 (35.7%)	58 (27.6%)		
≥15	206 (70.1%)	54 (64.3%)	152 (72.4%)		
No. of dissected LN stations				0.702	0.402
<6	91 (31.0%)	29 (34.5%)	62 (29.5%)		
≥6	203 (69.0%)	55 (65.5%)	148 (70.5%)		
Tumor grade				0.002	0.999
Well-differentiated	63 (21.4%)	18 (21.4%)	45 (21.4%)		
Moderately differentiated	136 (46.3%)	39 (46.4%)	97 (46.2%)		
Poorly differentiated	95 (32.3%)	27 (32.1%)	68 (32.4%)		
Adjuvant therapy				1.827	0.401
No	139 (47.3%)	41 (48.8%)	98 (46.7%)		
Chemotherapy	132 (44.9%)	34 (40.5%)	98 (46.7%)		
Radiotherapy/Chemoradiotherapy	23 (7.8%)	9 (10.7%)	14 (6.7%)		

*CT* computed tomography; *LN* lymph node

According to pre-and-postoperative CT findings, 84 patients (28.6%) were suspected to have metastatic unresected small LNs and 210 patients (71.4%) were considered as LN metastasis-negative. Among the 84 patients with suspected metastatic unresected small LNs, the number of supraclavicular, intrathoracic, abdominal, supraclavicular combined with intrathoracic, and intrathoracic combined with abdominal unresected small LNs was 14, 52, 3, 14, and 1, respectively. There were no significant differences in clinicopathologic characteristics between the CT-suspect and CT-negative groups (Table 1).

### Univariate analysis of the prognostic factors

**Fig. 2:**
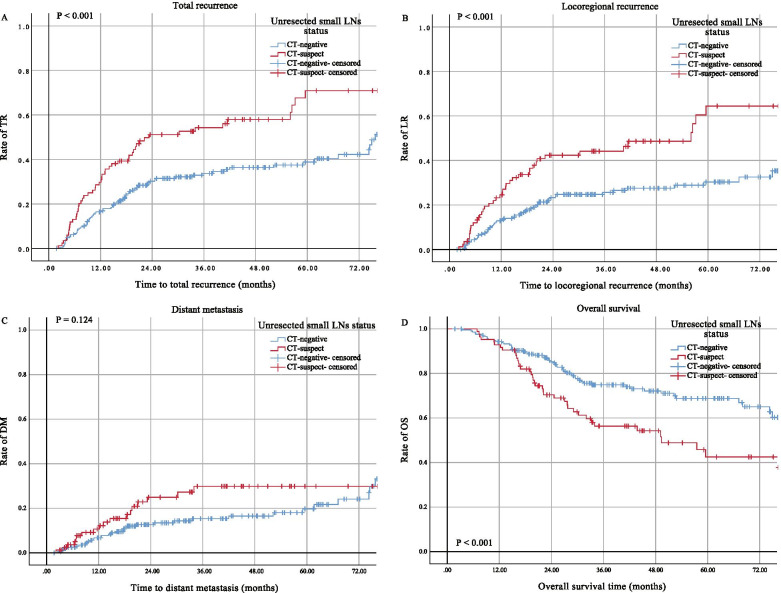
Kaplan-Meier curves for TR, LR, DM, and OS in patients with different unresected LNs status. TR, total recurrence; LR, locoregional recurrence; DM, distant metastasis; OS, overall survival; LN, lymph node; CT, computed tomography

Univariate analysis of the clinical and pathological data of patients showed that 3- and 5-year TR rates of the CT-suspect group were significantly higher than those of the CT-negative group (*P*<0.001) (Fig. 2A and Table 2). The univariate analysis also identified that the number of removed LNs, tumor grade, and postoperative adjuvant therapy were associated with TR (*P*<0.05) (Table 2). The 3- and 5-year LR rates of patients in the CT-suspect group were significantly higher than those of patients in the CT-negative group (*P* <0.001 (Fig. 2B and Table 2)). Furthermore, the univariate analysis showed that the number of removed LNs, tumor grade, and postoperative adjuvant therapy were associated with LR (*P*<0.05) (Table 2). However, there was no difference between the CT-suspect and CT-negative groups regarding the distant metastatic rate (*P*<0.124 (Fig. 2C and Table 2)). Only postoperative adjuvant therapy was identified to be associated with distant metastases (Table 2). The 3- and 5-year OS rates in the CT-suspect group were significantly lower than those in the CT-negative group (*P*<0.001 (Fig. 2D and Table 2)). In addition, the number of removed LNs, tumor grade, and postoperative adjuvant therapy were shown to be associated with OS through the univariate analysis (*P*<0.05) (Table 2).

**Table 2:** Univariate analysis of prognostic factors of 294 patients with pT3N0M0 thoracic esophageal squamous cell carcinoma

Variable	TR (%)		*χ*	*P*	LR (%)		*χ*	*P*	DM (%)		*χ*	*P*	OS (%)		*χ*	*P*
	3-year	5-year			3-year	5-year			3-year	5-year			3-year	5-year		
Sex			0.189	0.664			0.002	0.962			0.505	0.477			1.616	0.204
Male	40.7	48.5			31.0	40.1			20.4	21.8			67.7	58.3		
Female	36.4	47.0			30.6	39.4			14.6	25.9			75.1	71.2		
Age (years)			0.046	0.830			0.361	0.548			0.513	0.474			0.002	0.962
<60	40.3	44.1			34.0	38.2			16.8	19.1			67.7	62.3		
≥60	39.4	53.8			28.3	44.0			21.5	25.7			71.4	59.4		
Tumor location			2.180	0.336			1.485	0.476			2.570	0.277			1.092	0.579
Upper thoracic region	42.8	47.6			37.5	42.7			13.1	20.4			73.3	67.3		
Middle thoracic region	42.3	50.5			31.1	39.6			24.1	27.6			64.7	60.3		
Lower thoracic region	28.7	42.2			22.5	37.2			12.6	12.6			76.9	57.2		
Surgical approach			0.127	0.722			0.007	0.933			0.015	0.903			0.437	0.509
Ivor–Lewis	40.0	51.0			28.3	41.4			19.9	21.9			67.2	56.0		
McKeown	38.8	45.6			32.7	37.9			18.6	23.7			71.0	66.0		
Length of tumor			0.103	0.748			1.937	0.164			0.930	0.335			0.130	0.719
<4 cm	40.8	49.4			34.8	44.2			18.0	20.1			69.8	59.7		
≥4 cm	38.4	46.5			25.8	34.0			20.7	25.7			69.1	62.0		
Angioinvasion			0.011	0.916			0.048	0.826			0.725	0.394			0.005	0.945
No	39.9	47.7			30.9	40.4			19.0	22.6			69.6	60.8		
Yes	37.9	37.9			31.5	31.5			21.5	21.5			67.1	67.1		
Perineural invasion			0.004	0.947			0.063	0.802			0.092	0.761			1.010	0.315
No	39.3	48.9			30.7	40.9			19.0	22.9			71.4	61.9		
Yes	41.1	41.1			31.8	31.8			19.5	19.5			61.0	61.0		
No. of removed LNs			5.417	0.020*			4.866	0.027*			1.375	0.241			4.951	0.026*
<15	46.2	64.1			33.5	55.7			25.9	33.8			62.4	50.0		
≥15	36.9	40.0			29.7	32.2			16.4	17.7			72.4	66.4		
No. of dissected LN stations			2.335	0.126			1.827	0.176			0.888	0.346			1.718	0.190
<6	43.9	61.9			31.0	53.1			25.0	32.0			66.8	52.1		
≥6	37.8	40.2			30.9	32.4			16.5	17.9			70.5	66.8		
Tumor grade			8.299	0.016*			8.654	0.013*			4.274	0.118			7.385	0.025*
Well-differentiated	28.9	45.0			14.5	31.2			18.7	27.0			77.3	67.2		
Moderately differentiated	36.0	41.4			30.6	36.4			14.9	14.9			72.3	65.4		
Poorly differentiated	52.1	59.1			42.6	51.0			25.8	29.9			59.8	51.1		
Adjuvant therapy			20.708	<0.001*			18.589	<0.001*			6.774	0.034*			11.007	0.004*
No	54.8	66.1			46.7	58.0			25.7	38.1			60.1	48.6		
Chemotherapy	27.9	36.6			20.4	30.0			13.5	13.5			77.1	69.8		
Radiotherapy/chemoradiotherapy	24.3	35.1			9.3	22.3			20.7	32.0			73.8	63.3		
Unresected small LNs status			13.511	<0.001*			15.375	<0.001*			2.368	0.124			10.382	0.001*
CT-negative	33.7	38.8			25.6	30.4			15.3	18.7			74.8	68.8		
CT-suspect	54.3	70.9			44.1	64.4			29.9	29.9			56.3	42.5		

*TR* total recurrence; *LR* locoregional recurrence; *DM* distant metastasis; *OS* overall survival; *CT* computed tomography; *LN* lymph node

*P value < 0.05

### Multivariate analysis of the prognostic factors

The influencing factors for the prognosis of patients were placed in the Cox model for multivariate analysis. As shown in Table 3, unresected small LNs status, tumor grade, and postoperative adjuvant therapy were independent prognostic factors for TR, except the number of removed LNs. In addition, CT-suspect (HR=1.813; 95% CI, 1.263–2.602; *P*<0.001) and poorly differentiated (G3) tumor grade (HR=1.822; 95% CI, 1.118–2.968; *P*<0.016) were factors for higher TR. Both adjuvant chemotherapy (HR=0.466; 95% CI, 0.318–0.681; *P*<0.001) and aCRT/RT (HR=0.349; 95% CI, 0.160–0.763; *P*<0.008 ) were associated with lower TR. Unresected small LNs status, tumor grade, and postoperative adjuvant therapy were also found as independent prognostic factors for LR, and CT-suspect (HR=2.133; 95% CI, 1.407–3.233; *P*<0.001) and poorly differentiated (G3) tumor grade (HR=2.510; 95% CI, 1.334–4.723; *P*<0.004) were factors for higher LR. However, both POCT (HR=0.437; 95% CI, 0.279–0.683; *P*<0.001) and aCRT/RT (HR=0.256; 95% CI, 0.092–0.711; *P*<0.009) could reduce LR. Only postoperative adjuvant therapy was found as an independent prognostic factor for DM, and adjuvant chemotherapy was associated with lower DM (HR=0.462; 95% CI, 0.255–0.836; *P*<0.011). Unresected small LNs status, tumor grade, and postoperative adjuvant therapy were independent prognostic factors for OS of patients. CT-suspect (HR=1.807; 95% CI, 1.192–2.740; *P*<0.005) and poorly differentiated (G3) tumor grade (HR=1.878; 95% CI, 1.070–3.296; *P*<0.028) were factors for OS. Adjuvant chemotherapy was associated with higher OS (HR=0.516; 95% CI, 0.322–0.703; *P*<0.003), but there was no association between aCRT/RT (HR=0.564; 95% CI, 0.255–1.248; *P*<0.158) and OS.

**Table 3:** Multivariate analysis of prognostic factors of 294 patients with pT3N0M0 thoracic esophageal squamous cell carcinoma

Item	P	HR	95% CI	
			Lower	Upper
**TR**				
No. of removed LNs				
<15		Reference		
≥15	0.065	0.708	0.491	1.022
Tumor grade	0.033*			
Well-differentiated		Reference		
Moderately differentiated	0.368	1.253	0.767	2.050
Poorly differentiated	0.016*	1.822	1.118	2.968
Adjuvant therapy	<0.001*			
No		Reference		
Chemotherapy	<0.001*	0.466	0.318	0.681
Radiotherapy/chemoradiotherapy	0.008*	0.349	0.160	0.763
Unresected small LNs status				
CT-negative		Reference		
CT-suspect	0.001*	1.813	1.263	2.602
**LR**				
No. of removed LNs				
<15		Reference		
≥15	0.061	0.665	0.434	1.019
Tumor grade	0.017*			
Well-differentiated		Reference		
Moderately differentiated	0.046*	1.900	1.011	3.569
Poorly differentiated	0.004*	2.510	1.334	4.723
Adjuvant therapy	<0.001*			
No		Reference		
Chemotherapy	<0.001*	0.437	0.279	0.683
Radiotherapy/chemoradiotherapy	0.009*	0.256	0.092	0.711
Unresected small LNs status				
CT-negative		Reference		
CT-suspect	<0.001*	2.133	1.407	3.233
**DM**				
Adjuvant therapy	0.039*			
No		Reference		
Chemotherapy	0.011*	0.462	0.255	0.836
Radiotherapy/chemoradiotherapy	0.423	0.675	0.259	1.764
**OS**				
No. of removed LNs				
<15		Reference		
≥15	0.109	0.708	0.464	1.080
Tumor grade	0.043*			
Well-differentiated		Reference		
Moderately differentiated	0.565	1.181	0.670	2.083
Poorly differentiated	0.028*	1.878	1.070	3.296
Adjuvant therapy	0.010*			
No		Reference		
Chemotherapy	0.003*	0.516	0.332	0.803
Radiotherapy/chemoradiotherapy	0.158	0.564	0.255	1.248
Unresected small LNs status				
CT-negative		Reference		
CT-suspect	0.005*	1.807	1.192	2.740

*TR* total recurrence; *LR* locoregional recurrence; *DM* distant metastasis; *OS* overall survival; *CT* computed tomography; *LN* lymph node

*P value < 0.05

### Recurrence pattern

A total of 127 out of 294 patients experienced recurrence by the end of follow-up. Among them, 74 (25.2%) had LR, 34 (11.6%) had DM, and 19 (6.5%) had LR with DM (the relationship between local recurrence and CT-suspect metastatic unresected small lymph nodes are shown in Appendix (Table 2)). Overall, the rates of TR and LR in the CT-suspect group were significantly higher than that in the CT-negative group (TR: *P*<0.001; LR: *P*<0.001 (Table 4)). The rate of DM in the CT-suspect group was higher than that in the CT-negative group, but the difference was not significant (*P*<0.337 (Table 4)). Among the LRs, the rate of supraclavicular LN recurrence in the CT-suspect group was significantly higher than that in the CT-negative group (*P*<0.001 (Table 4)).

**Table 4:** Causes of treatment failure in different groups

No. of patients experiencing failure (%)					
Disease recurrence		CT-suspect group (*n*=84)	CT-negative group (*n*=210)	*χ*^2^ value	*P* value
Total		50 (59.5%)	77 (36.7%)	12.775	<0.001*
Local		40 (47.6%)	53 (25.2%)	13.897	<0.001*
	Sup.LN	19 (22.6%)	10 (4.8%)	21.519	<0.001*
	Med.LN	17 (20.2%)	37 (17.6%)	0.274	0.600
	Abd.LN	5 (6%)	5 (2.4%)	1.369	0.155
	Tumor bed	2 (2.4%)	4 (1.9%)	0.000	1.000
	Ana	4 (4.8%)	2 (1.0%)	2.658	0.058
Metastasis		18 (21.4%)	35 (16.7%)	0.921	0.337

*CT* computed tomography; *Sup* supraclavicular; *Med* mediastinal; *Abd* abdominal; *Ana* anastomotic; *LN* lymph node

*P value < 0.05

## Discussion

In this study, we first emphasized the prognostic significance of unresected small LNs assessment for patients with pT3N0M0 TESCC who received esophagectomy. Univariate analysis identified the number of removed LNs, tumor grade, postoperative adjuvant therapy, and unresected small LNs status as prognostic factors (*P* < 0.05). However, the results of multivariate factor analyses showed that tumor grade, postoperative adjuvant therapy, and unresected small LNs status are independent prognostic factors (*P* < 0.05). Further analysis showed unresected small LNs in CT-suspect status shown a higher TR (*P*<0.001) and LR (*P*<0.001) rates compared with unresected small LNs in CT-negative status.

For the high lymph node spread of TESCC, the long-term survival of a patient is highly dependent on the extent of lymphadenectomy [17, 18]. Previous studies have proven that an increased extent of lymphadenectomy is associated with improved survival [19, 20]. Thus, a wide range of thresholds ranging from 6 to 20 has been reported as the optimum number of removed LNs for patients with pT3N0M0 TESCC in previous studies [21–24]. However, several studies found no association between the number of removed LNs and improved survival in TESCC cases [25–28]. In this study, we used 15 LNs as a threshold to analyze the prognostic value of the number of removed LNs in patients with pT3N0M0 TESCC, as recommended by the current National Comprehensive Cancer Network guidelines [29]. The number of removed LNs was a significant risk factor for survival in the univariate analysis but not in the multivariate analysis. Evidence indicates that the survival benefits from a higher number of removed LNs can be partly attributed to stage migration (improved staging rather than improved therapeutic benefit of the dissection itself) [30–32]. With a higher number of removed LNs, the possibility of discovering potential cancer-positive LNs will be improved, allowing more accurate staging and treatment protocols. This indicates that metastatic nodes may be among the unresected LNs.

Additionally, setting a unified threshold to represent optimum lymphadenectomy seems unreasonable for patients with different numbers of LNs before surgery. Further, overtreatment may result in increased complications and mortality, and owing to the limitations of surgical skills and the patient’s physical condition, there may still be unresected lymph nodes. Hence, the precise evaluation of unresected LNs for cancer infiltration was not only the primary determinant of accurate residual tumor (R) classification [11], but also meaningful for prognosis predicting and postoperative decision-making and management of EC [5].

Esophageal CT is currently the most commonly used method for lymph node assessment. However, the accuracy of CT is unsatisfactory when LNs greater than 10-mm diameter are considered positive for metastasis [33, 34]. Wakita et al. [35] found that among the 213 patients who were diagnosed as cN0 by CT and underwent curative esophagectomy without preoperative neoadjuvant treatment, 60 (28%) patients had LN metastasis diagnosed pathologically. An important limitation was that metastatic lymph nodes might present without an apparent enlargement in size. Furthermore, some enlarged nodes may contain no metastasis. Past reports have shown that the accuracy of CT in the diagnosis of lymph node metastasis can be 46–58% [36], and a false-negative rate of 11–56% was reported [37]. Even with PET/CT, a large number of small metastatic lesions can be difficult to detect [33, 38, 39]. A previous study has reported that lowering the size criteria and combining the axial ratio (short-axis diameter/long-axis diameter) would increase sensitivity [16, 35, 40]. Therefore, in the present study, LNs were diagnosed as suspected metastatic nodes in the absence of pathological confirmation, when the short-axis diameter exceeding 5 mm and the shape of the node was round (axial ratio exceeding 0.66). However, further research is needed to determine the most accurate diagnostic method.

We retrospectively examined 294 patients with stage pT3N0M0 TESCC who received radical esophagectomy between 2009 and 2017. Among them, 84 patients (42.5%) were suspected to have metastatic unresected lymph nodes evaluated using pre- and postoperative CT and were classified into the CT-suspect group. The CT-suspect group showed significantly higher TR and LR than the CT-negative group. Further analysis found that the presence of suspected metastatic unresected small LNs was an independent prognostic factor for TR, LR, and OS. Therefore, compared with the number of removed LNs, unresected small LNs with suspected metastasis may be a better reflector for the thoroughness of lymphadenectomy and a more important prognostic factor for pT3N0M0 TESCC.

In addition, in the overall study cohort, the TR rate among pT3N0M0 thoracic ESCC patients was as high as 43.2%, and the LR, DM, and LR with DM rates were 25.2, 11.6, and 6.5%, respectively. These results are consistent with the previous findings [25, 41]. Further analysis showed that the most common area for LR was different in CT-suspect group and CT-negative group. In contrast to previous studies [25], the supraclavicular lymphatic area was the most common area for LR in the CT-suspect group with a recurrence rate of 22.6%, but the mediastinal lymphatic area was the most common area for LR in the CT-negative group with a recurrence rate of 17.6%. We believe that this might be owing to the high proportion of unresected small LNs in the supraclavicular lymphatic area in the CT-suspect group. These findings suggested that CT-suspect metastatic lymph nodes should be removed as extensively as possible during the operation, or the major postoperative failure areas, such as the supraclavicular lymphatic recurrence area, and the mediastinal lymphatic recurrence area should be carefully included in the clinical target area during postoperative radiotherapy.

Currently, grade differentiation (G categories) remains an important parameter for pathologic staging of pT3N0M0 thoracic esophageal squamous cell carcinoma in the latest 8th edition of the AJCC TNM staging classification for esophageal and esophagogastric cancers [42]. In this study, we found that tumor differentiation was a prognostic factor for thoracic pT3N0M0 ESCC. Patients with moderate to well-differentiated tumors had a better prognosis than those with poorly differentiated tumors. This is consistent with previous studies [25]. This is probably because poorly differentiated ECs may have a higher lymph node metastasis rate, regional recurrence rate, and distant metastasis rate. Zhou et al. [43] reported that the presence of the poor tumor differentiation (*p* < 0.05) was an independent predictor for lymph node metastasis in superficial esophageal squamous carcinoma patients. Liu et al. [44] reported that tumor differentiation was an independent risk factor for regional lymphatic recurrence. The distant metastasis rate of poorly differentiated EC was significantly higher than that of moderately and well-differentiated EC [44, 45]. However, Situ et al. [21] retrospectively analyzed 302 patients with postoperative pathologic stage T3N0M0 who underwent esophagectomy and found that histologic tumor grade had no significant influence on patient survival. The variations in study findings are probably due to the proportion of patients with poorly differentiated tumors, postoperative treatment protocols, and the extent of lymphadenectomy.

Additionally, there are still controversies about the value and pattern of postoperative adjuvant therapy in pT3N0M0 TESCC patients. Thus, the use of postoperative chemotherapy or radiation in pT3N0M0 cases needs to be characterized in more detail, including the presence of suspicious unresected small LNs. Our previous study compared surgery alone and POCT using a propensity score matching (PSM) analysis for 582 patients with pT3N0M0 TESCC, retrospectively. After PSM, both groups had similar factors. Surgery + POCT significantly improved the 5-year OS and DFS (OS, 70.8% vs. 52.8%, *P*<0.0001; DFS, 66.5% vs. 50.2%, *P*<0.0001) [24]. However, other studies have shown no survival benefit for patients who received POCT. Ando et al. [46] reported no survival benefit of 5-year OS for the Japan Clinical Oncology Group, with a 5-year OS rate of 48.1% for POCT and 44.9% for surgery alone (*P*=0.26). For N1 ESCC patients, the 5-year OS was 43.7% for patients of POCT and 35.5% in surgery alone (*P*=0.15). The value of adjuvant PORT in pT3N0M0 TESCC patients is also controversial; this may be related to inclusion criteria, surgical methods, extent of exposure, and radiotherapy technologies. Yang et al. [22] conducted a large sample-size retrospective study of PORT for pT3N0M0 TESCC, which showed that, compared with surgery alone, surgery + PORT significantly improved the 5-year OS (75.2% vs. 58.5%, *P*=0.004) and DFS (71.8% vs. 49.2%, *P*=0.001) rates. However, in other reports, no benefit of survival was observed. Xiao et al. [47] conducted a large phase III clinical trial of PORT in esophageal carcinoma and showed an improved 3-year OS in patients with pT2–3N0M0 TESCC who received PORT (64.0%) versus those who underwent surgery alone (56.0%); however, the difference was not significant. One possible reason is the inaccuracy of N staging. In most studies that show no survival benefit from postoperative adjuvant treatment, the most common surgical approach is left thoracotomy or two-field esophagectomy. Previous studies have demonstrated that LN dissection is more complete using right thoracotomy than left thoracotomy, especially for tracheoesophageal groove and para-recurrent laryngeal nerve LNs [48, 49], although transcervical video-assisted mediastinoscopic lymphadenectomy (VAMLA) via the left thoracic approach may avoid this situation, but it is not widely used [50]. Cervical LNs were seldom resected during two-field esophagectomy. LNs in the upper mediastinum (especially above the arch of the aorta) were usually dissected incompletely. Therefore, potential metastasis of LNs in the cervical region and upper mediastinum could not be removed intraoperatively in these studies. Therefore, postoperative chemotherapy may be insufficient; on the contrary, postoperative radiotherapy may be more effective at this time. Wang et al. [23] reported that postoperative adjuvant therapy (most of the patients received POCT) was not associated with OS (*P*>0.05), but the presence of small LNs on preoperative CT was an independent prognostic factor for OS. In their study, all or most patients with small LNs on CT located in the upper mediastinum (especially above the arch of the aorta) underwent a limited left thoracotomy in a two-field esophagectomy; thus, these small LNs may be unresected after surgery, indicating that no benefit of postoperative adjuvant therapy was observed in this particular study because of the unresected lymph nodes. These results suggest that unresected small LNs assessment for patients with pT3N0M0 TESCC receiving esophagectomy may be important for further postoperative treatment decisions. However, the best pattern and indicator of postoperative adjuvant therapy needs to be further studied in pT3N0M0 TESCC.

## Conclusions

In summary, this study revealed that postoperative unresected LN assessment for ESCC patients is critically important. The unresected small LNs status is the main influencing factor for prognosis and recurrence, and the recurrence rate of patients with unresected small LNs in CT-suspect metastasis was significantly higher than that in CT-negative EC. Therefore, CT-suspect metastatic lymph nodes should be removed as extensively as possible during the operation, and the optimal postoperative treatment may consider the status of unresected small LNs which assessed by pre- and postoperative CT.

## Supplementary Information

**Additional file 1.**

## Data Availability

Not applicable

## Abbreviations

EC: Esophageal cancer
ESCC: Esophageal squamous cell carcinoma
TESCC: Thoracic esophageal squamous cell carcinoma
R0 resection: Radical resection
LNs: Lymph nodes
AJCC: American Joint Committee on Cancer
TNM: Tumor–Node–Metastasis
POCT: Postoperative adjuvant chemotherapy
aCRT/RT: Adjuvant chemoradiotherapy/radiotherapy
IMRT: Intensity-modulated radiotherapy
CT: Computed tomography
TR: Total recurrence
LR: Locoregional recurrence
DM: Distant metastasis
OS: Overall survival
HR: Hazard ratio
CI: Confidence interval
DFS: Disease-free survival
