# Supplementary material for: Unresected small lymph node assessment predicts prognosis for patients with pT3N0M0 thoracic esophageal squamous cell carcinoma
Source: World J Surg Oncol. 2021 Oct 18;19:303. doi: 10.1186/s12957-021-02412-1 (PMC8522218; doi:10.1186/s12957-021-02412-1)
Supplement: Supplementary file 1 — Additional file 1. [file 12957_2021_2412_MOESM1_ESM.docx]

Table 1. The size change of CT-suspect metastatic unresected small Lymph Nodes in different conditions

| Adjuvant therapy | Tumor grade | No. of unresected small Lymph Nodes in CT-suspect | Changes in lymph nodes size after surgery（No. of patient） | | Total | Changes in lymph node size after adjuvant treatment （No. of patient） | | | | Total |
| --- | --- | --- | --- | --- | --- | --- | --- | --- | --- | --- |
|  |  |  | No change | Increase |  | No change | Increase | Shrink | Uncertain |  |
| No | G1 | 1 | 6 | 1 | 7 |  |  |  |  |  |
|  |  | 2 | 1 |  | 1 |  |  |  |  |  |
|  |  | 4 | 1 |  | 1 |  |  |  |  |  |
|  |  | Total | 8 | 1 | 9 |  |  |  |  |  |
|  | G2 | 1 | 6 | 6 | 12 |  |  |  |  |  |
|  |  | 2 | 2 | 4 | 4 |  |  |  |  |  |
|  |  | 5 |  | 1 | 1 |  |  |  |  |  |
|  |  | 6 | 1 |  | 1 |  |  |  |  |  |
|  |  | Total | 9 | 11 | 2 |  |  |  |  |  |
|  | G3 | 1 | 7 | 2 | 9 |  |  |  |  |  |
|  |  | 2 | 2 |  | 2 |  |  |  |  |  |
|  |  | 3 |  | 1 | 1 |  |  |  |  |  |
|  |  | Total | 9 | 3 | 12 |  |  |  |  |  |
|  | Total |  | 26 | 15 | 41 |  |  |  |  |  |
| Chemotherapy | G1 | 1 | 3 |  | 3 | 2 | 1 |  |  | 3 |
|  |  | 2 | 4 |  | 4 | 3 | 1 |  |  | 4 |
|  |  | 4 | 1 |  | 1 | 1 |  |  |  | 1 |
|  |  | Total | 8 |  | 8 | 6 | 2 |  |  | 8 |
|  | G2 | 1 | 6 | 1 | 7 | 5 | 2 |  |  | 7 |
|  |  | 2 | 3 | 1 | 4 | 1 | 2 | 1 |  | 4 |
|  |  | 5 | 2 |  | 2 | 2 |  |  |  | 2 |
|  |  | Total | 11 | 2 | 13 | 8 | 4 | 1 |  | 13 |
|  | G3 | 1. | 7 | 3 | 1 | 5 | 4 |  | 1 | 1 |
|  |  | 2. |  | 1 | 1 |  | 1 |  |  | 1 |
|  |  | 3. | 2 |  | 2 | 1 |  |  | 1 | 2 |
|  |  | Total | 9 | 4 | 13 | 6 | 5 |  | 2 | 13 |
|  | Total |  | 28 | 6 | 34 | 20 | 11 | 1 | 2 | 34 |
| Radiotherapy/Chemoradiotherapy | G1 | 2 | 1 |  | 1 | 1 |  |  |  | 1 |
|  | G2 | 1 | 3 |  | 3 | 3 |  |  |  | 3 |
|  |  | 2 | 2 | 1 | 3 | 1 | 1 | 1 |  | 3 |
|  |  | Total | 5 | 1 | 6 | 4 | 1 | 1 |  | 6 |
|  | G3 | 1 | 2 |  | 2 | 2 |  |  |  | 2 |
|  | Total |  | 8 | 1 | 9 | 7 | 1 | 1 |  | 9 |
| Total |  |  | 62 | 22 | 84 | 27 | 12 | 2 | 2 | 43 |

Note: CT: Computed tomography; G1: Well-differentiated; G2: Moderately differentiated; G3: Poorly differentiated.

Table 2. The relationship between local recurrence and CT-suspect metastatic unresected small Lymph Nodes

| Adjuvant therapy | Tumor grade | No. of unresected small Lymph Nodes in CT-suspect | No. of patients with local recurrence | Whether the local recurrence is caused by CT-suspect metastatic unresected small Lymph Nodes | | |
| --- | --- | --- | --- | --- | --- | --- |
|  |  |  |  | No | Yes | Uncertain* |
| No | G1 | 1 | 1 |  | 1 |  |
|  |  | Total | 1 |  | 1 |  |
|  | G2 | 1 | 11 | 1 | 6 | 4 |
|  |  | 2 | 4 |  | 4 |  |
|  |  | 5 | 1 |  | 1 |  |
|  |  | Total | 16 | 1 | 11 | 4 |
|  | G3 | 1 | 3 | 1 | 2 |  |
|  |  | 3 | 1 |  | 1 |  |
|  |  | Total | 4 | 1 | 3 |  |
|  | Total |  | 21 | 2 | 15 | 4 |
| Chemotherapy | G1 | 1 | 2 | 1 | 1 |  |
|  |  | 2 | 2 | 1 | 1 |  |
|  |  | Total | 4 | 2 | 2 |  |
|  | G2 | 1 | 2 |  | 2 |  |
|  |  | 2 | 3 |  | 3 |  |
|  |  | Total | 5 |  | 5 |  |
|  | G3 | 1 | 6 | 1 | 5 |  |
|  |  | 2 | 1 |  | 1 |  |
|  |  | 3 | 1 |  |  | 1 |
|  |  | Total | 8 | 1 | 6 | 1 |
|  | Total |  | 17 | 3 | 13 | 1 |
| Radiotherapy/Chemoradiotherapy | G2 | 2. | 2 |  | 2 |  |
|  | Total |  | 2 |  | 2 |  |
| Total |  |  | 40 | 5 | 30 | 5 |

Note: CT: Computed tomography; G1: Well-differentiated; G2: Moderately differentiated; G3: Poorly differentiated; *: There were 4 cases in the non-adjuvant treatment group and 1 case in the adjuvant chemotherapy group whose local recurrence was diagnosed in the local hospital. We only have the diagnosis report but no CT images, so the relationship between the local recurrence and CT-suspect metastatic unresected small Lymph Nodes cannot be judged.
